# Bilateral Quadriceps Tendon Rupture Complicated by Bilateral Postoperative Septic Arthritis: A Case Report

**DOI:** 10.7759/cureus.113369

**Published:** 2026-07-25

**Authors:** Karla Andrade de Oliveira, Ashley Cardona, Mirela Ponduchi

**Affiliations:** 1 Internal Medicine, Universidad Autónoma de Guadalajara (UAG) School of Medicine, Guadalajara, MEX; 2 Radiology, Universidad Autónoma de Guadalajara (UAG) School of Medicine, Guadalajara, MEX; 3 Internal Medicine, Abrazo Community Health Network, Phoenix, USA

**Keywords:** quadriceps tendon, radiology, septic arthritis, surgery, tendon rupture

## Abstract

Quadriceps tendon rupture (QTR) is a rare injury, largely due to the tendon’s ability to withstand substantial tensile forces under normal physiological conditions. When it occurs, it is typically associated with systemic risk factors such as chronic kidney disease, diabetes mellitus, or prolonged corticosteroid use. QTR results in significant functional impairment, and prompt surgical repair is essential to restore mobility. Septic arthritis is an uncommon postoperative complication that typically affects a single joint and can rapidly destroy articular cartilage if not identified and treated early. We present a rare case of bilateral QTR complicated by bilateral septic arthritis following surgical repair, emphasizing the importance of early diagnosis and aggressive management of surgical site infections to prevent permanent joint damage and prolonged disability.

## Introduction

The quadriceps tendon connects the quadriceps muscle group to the patella, playing a critical role in knee extension and lower limb function. This enables activities such as walking, running, jumping, and rising from a seated position. It also contributes to patellar stability during knee movement [1]. Quadriceps tendon rupture (QTR) is a rare but serious injury that typically occurs unilaterally and more frequently in males, with a male-to-female ratio of approximately 4.2:1 [2]. It most commonly affects individuals over the age of 40, with peak incidence in the fifth and sixth decades of life [1,3]. Bilateral QTR is exceptionally rare, accounting for fewer than 3% of all cases [4]. 

Rupture is often precipitated by a sudden forceful contraction or mechanical overload but rarely occurs in the absence of underlying predisposing factors. Common risk factors include systemic diseases such as chronic kidney disease, diabetes mellitus, rheumatoid arthritis, hyperparathyroidism, and gout, medication use including long-term corticosteroids and fluoroquinolones, and age-related tendon degeneration [1,3,5-7]. 

While surgical repair is the standard treatment, it carries inherent risks, including infection. Septic arthritis following tendon repair is a rare but serious complication that can result in joint destruction, prolonged recovery, or permanent disability [8,9]. The most common causative organisms are Staphylococcus aureus, including methicillin-resistant *Staphylococcus aureus* (MRSA), and *Streptococcus *species [9]. Postoperative septic arthritis typically presents within two to four weeks of surgery and requires prompt recognition and treatment [8,9]. Bilateral postoperative septic arthritis following bilateral QTR is exceptionally rare, and no published studies have established its incidence.

This report describes a 57-year-old African American male who presented with severe bilateral knee pain. Magnetic resonance imaging (MRI) revealed bilateral QTR, prompting immediate surgical repair. Despite an initially favorable postoperative course, the patient subsequently developed bilateral septic arthritis. This case highlights the importance of early diagnosis and vigilant monitoring for postoperative complications.

## Case presentation

We report the case of a 57-year-old African American male patient who presented to the emergency department after falling down a flight of stairs and landing directly on both knees. He experienced immediate, severe bilateral knee pain and was unable to bear weight on either lower extremity. Family members found him in distress and called emergency medical services.

His medical history was significant for hypertension, hyperlipidemia, and gastroesophageal reflux disease, for which he was taking losartan 100 mg, atorvastatin 80 mg, and pantoprazole 40 mg. His social history was notable for a 20-pack-year smoking history and occasional alcohol consumption, consisting of approximately two beers once per month. He denied illicit drug use and had no prior surgical history.

On presentation, laboratory evaluation demonstrated a white blood cell (WBC) count of 9.8 ×10³/µL, hemoglobin of 14.2 g/dL, platelet count of 275 ×10³/µL, erythrocyte sedimentation rate (ESR) of 18 mm/hr, C-reactive protein (CRP) of 6 mg/L, and hemoglobin A1c of 5.6%. The comprehensive metabolic panel was unremarkable, with preserved renal function (creatinine 0.9 mg/dL) and normal electrolyte levels. 

**Figure 1:**
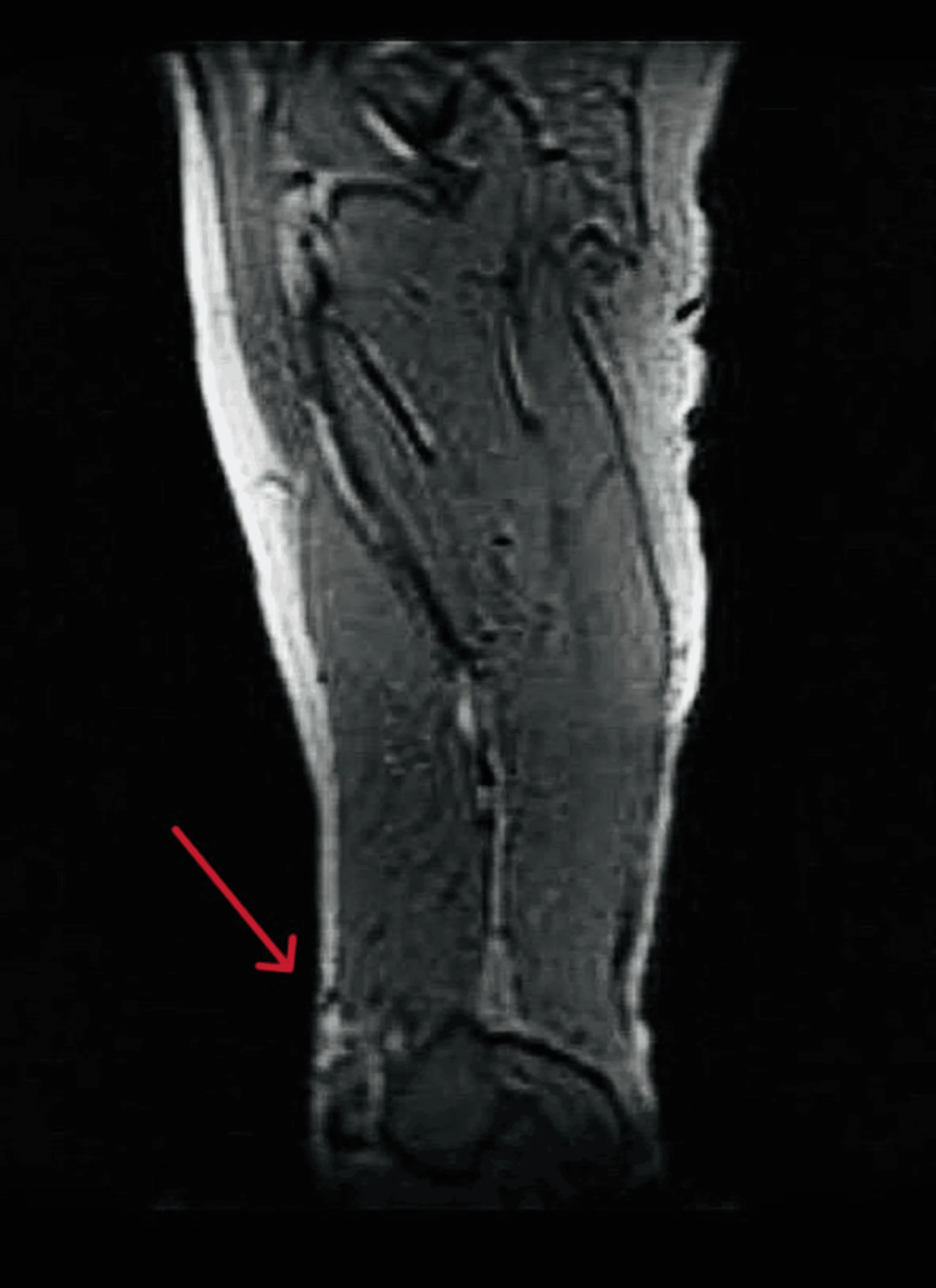
Sagittal T2-weighted MRI of the left knee demonstrating discontinuity of the quadriceps tendon at its insertion on the superior pole of the patella (arrow).

**Figure 2:**
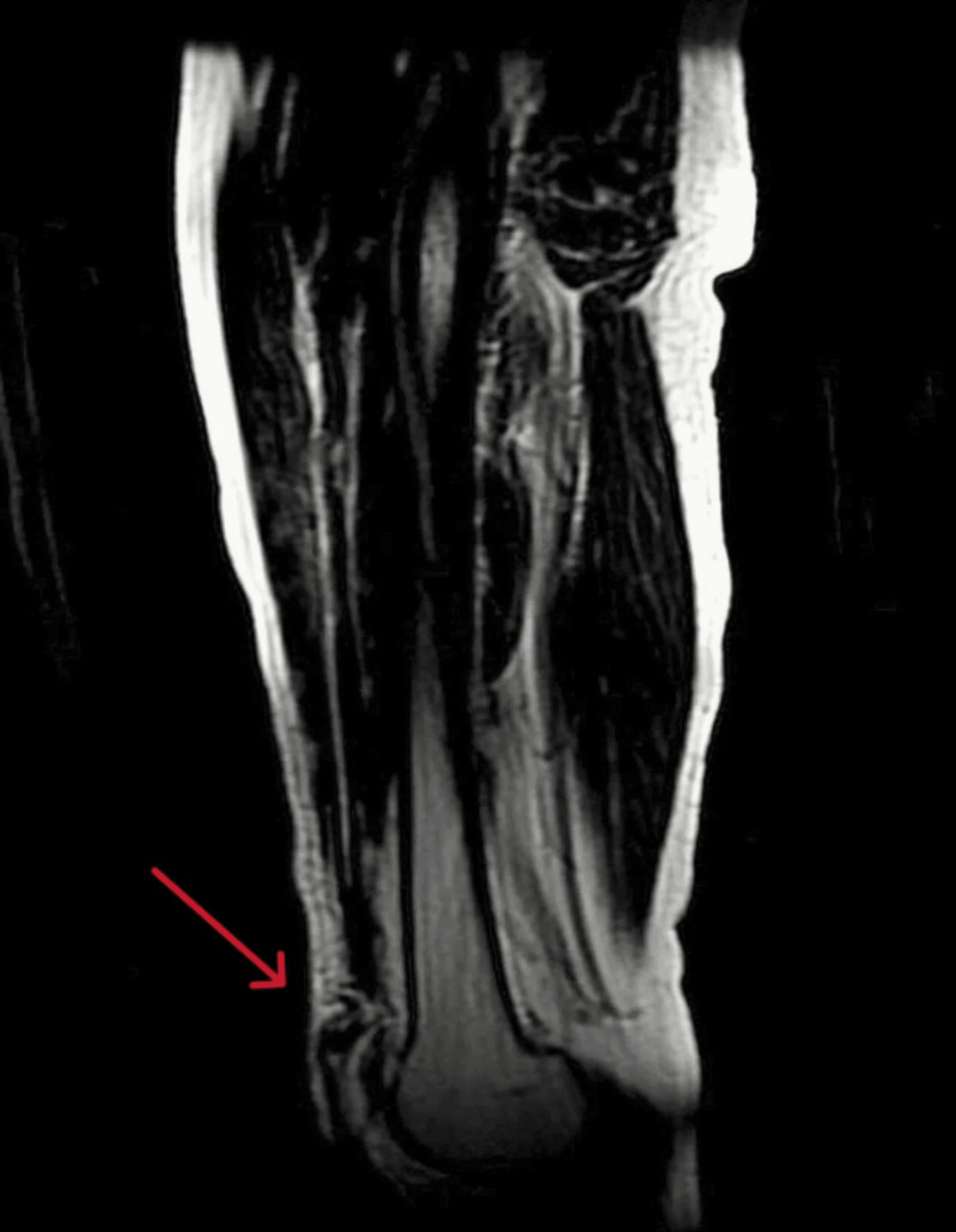
Sagittal T2-weighted MRI of the right knee demonstrating discontinuity of the quadriceps tendon at its insertion on the superior pole of the patella (arrow).

Bilateral knee MRI demonstrated no acute fracture or dislocation (Figure 1). MRI revealed high-grade near-full-thickness quadriceps tendon tears at the patellar attachment sites bilaterally (Figure 2). The patient subsequently underwent surgical repair on hospital day 1. Intraoperatively, complete bilateral QTRs with medial and lateral retinacular tears were identified. A four-strand FiberWire (Arthrex, Inc., Naples, FL, USA) repair was performed on both knees.

**Figure 3:**
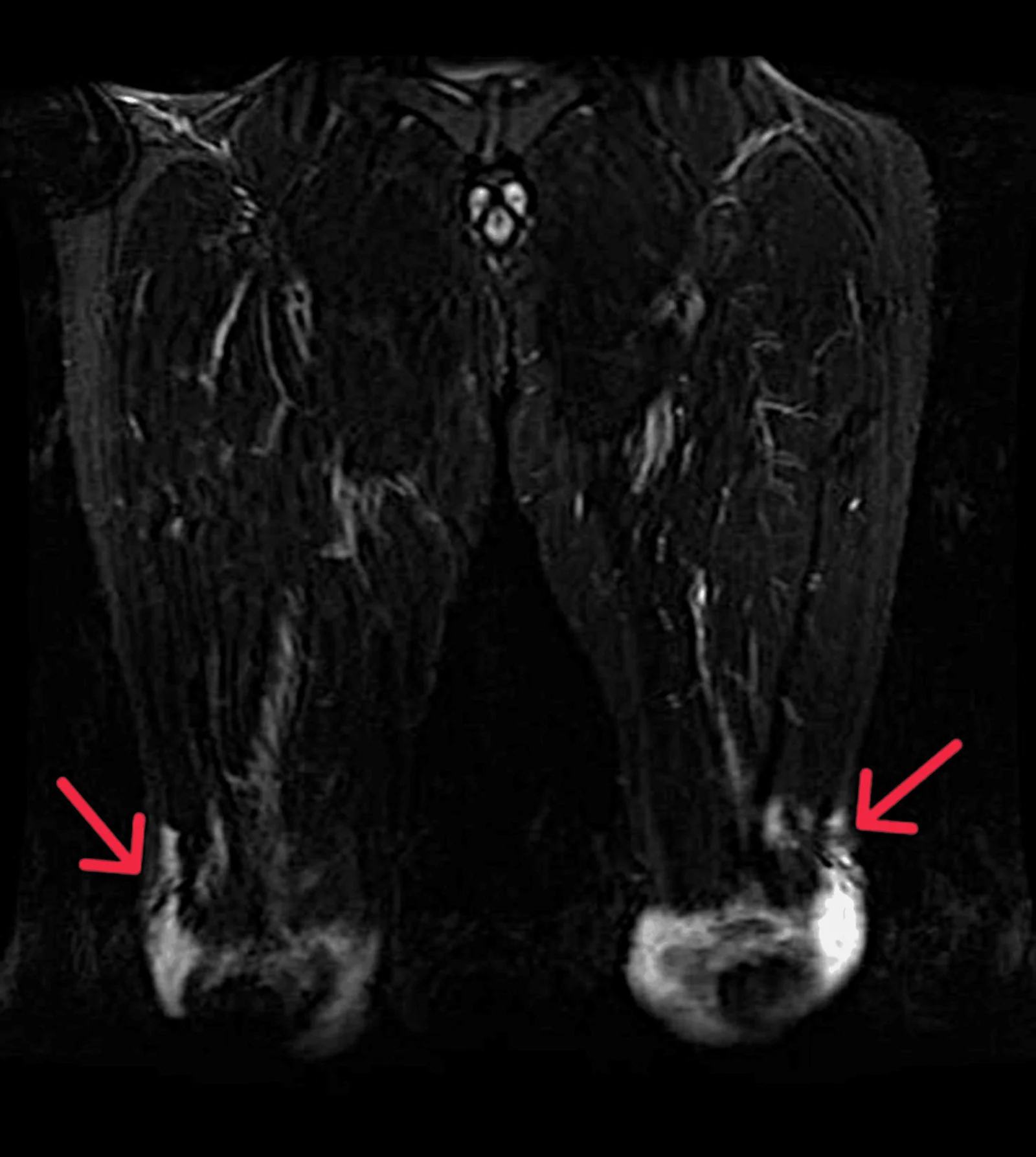
Coronal T2-weighted MRI of both knees demonstrating bilateral complete quadriceps tendon ruptures at the superior poles of the patellae, with tendon retraction and surrounding soft-tissue edema (arrows).

The initial postoperative course was uncomplicated, and the patient was discharged to a skilled nursing facility. Approximately two weeks after surgery (postoperative day 14), he returned to the emergency department with severe bilateral knee pain and swelling, more pronounced on the right side. He was febrile (38.6°C (101.5°F)), and laboratory studies demonstrated leukocytosis (WBC of 16.5 ×10³/µL), elevated ESR (82 mm/hr), and elevated CRP (195 mg/L), consistent with an inflammatory and infectious process. MRI demonstrated bilateral suprapatellar effusions (Figure 3). Arthrocentesis was performed on both knees. Aspiration of the right knee yielded approximately 30 mL of seropurulent fluid, while aspiration of the left knee yielded approximately 20 mL of cloudy synovial fluid. Synovial fluid analysis demonstrated a nucleated cell count of approximately 96,000 cells/µL (94% neutrophils) in the right knee and 82,000 cells/µL (91% neutrophils) in the left knee. Gram stain from both aspirates demonstrated gram-positive cocci in clusters, and synovial fluid cultures subsequently grew methicillin-sensitive *Staphylococcus aureus* (MSSA). Blood cultures remained negative. The patient subsequently underwent operative irrigation and debridement. Intraoperative evaluation confirmed rerupture of the quadriceps tendon at the superior pole of the patella. Although the FiberWire sutures remained intact, the presence of infected wound beds and tendon retraction precluded immediate revision. Extensive irrigation and debridement of both knees was therefore performed.

Three weeks after the initial irrigation and debridement, the patient developed progressive erythema, drainage, and tenderness. Repeat laboratory evaluation demonstrated persistent leukocytosis (WBC 14.8 × 10³/µL) and elevated inflammatory markers (ESR 98 mm/hr, CRP 168 mg/L), consistent with ongoing infection. Due to persistent bilateral septic arthritis, he underwent a third operation consisting of bilateral partial synovectomy. Intraoperatively, the right knee demonstrated mild synovitis, whereas the left knee contained minimal fluid and cellulitic changes without gross purulence. Synovial fluid and intraoperative tissue cultures obtained from both knees grew MSSA, confirming bilateral postoperative septic arthritis. The patient was initially treated empirically with intravenous vancomycin and cefepime. Following infectious disease consultation and antimicrobial susceptibility testing, therapy was narrowed to intravenous cefazolin. He remained febrile during the first three postoperative days but demonstrated gradual clinical improvement thereafter. After stabilization, he was discharged to a skilled nursing facility for continued intravenous antibiotic therapy, rehabilitation, and assistance with mobility and activities of daily living. 

## Discussion

QTR is an uncommon but functionally devastating injury, representing fewer than 2% of all tendon ruptures. It primarily affects men older than 40 years and is usually unilateral [3,9]. Bilateral QTR is particularly rare, accounting for less than 3% of quadriceps tendon injuries [4]. The typical mechanism involves a sudden, forceful contraction of the quadriceps muscle against a flexed knee, often during a fall or abrupt deceleration [3]. Several systemic conditions have been identified as predisposing factors, including chronic kidney disease, diabetes mellitus, hyperparathyroidism, gout, and rheumatologic disorders [1,3,7,10]. Long-term corticosteroid and fluoroquinolone use has also been associated with an increased risk of rupture [2,10]. Histopathologic examination of ruptured tendons commonly demonstrates degenerative changes, including myxoid degeneration, collagen disorganization, and hypovascularity [3,6,10]. These changes may be subclinical and progressive, weakening the tendon's structural integrity over time. 

Surgical repair remains the standard treatment for complete QTR. Prompt surgical repair is recommended, as delays may result in tendon retraction, scar formation, and deterioration of tendon quality, thereby complicating repair and reducing the likelihood of full functional recovery [3,11]. In a study of 66 patients who underwent QTR repair, Langenhan et al. reported that early functional mobilization combined with full weight-bearing was safe and produced outcomes comparable to those achieved with immobilization and protected weight-bearing [12]. Although most patients regain satisfactory function after repair, some may experience persistent weakness or limited knee flexion [5].

Postoperative infection following tendon repair is relatively rare. When infection extends into the joint space, it can result in septic arthritis, a rapidly destructive condition requiring urgent intervention [13]. Septic arthritis is most commonly caused by hematogenous spread or direct inoculation during procedures and is typically monoarticular [9]. The most commonly isolated pathogens include *Staphylococcus aureus*, including MRSA, and *Streptococcus *species [9,14]. Risk factors for postoperative septic arthritis include immunosuppression, diabetes mellitus, obesity, advanced age, and preexisting joint disease [9,14]. Several of these comorbidities have also been implicated in tendon weakening, suggesting a shared pathophysiologic susceptibility. Although our patient lacked many of these established systemic risk factors, postoperative infection remains a recognized complication of surgery despite adherence to accepted sterile technique. No evidence of intraoperative contamination was identified in this case. Nevertheless, postoperative septic arthritis should remain an important diagnostic consideration in any patient presenting with persistent pain, erythema, drainage, or fever following tendon repair. Prompt recognition, appropriate perioperative antibiotic prophylaxis, meticulous sterile technique, careful wound management, and timely surgical irrigation and debridement are critical to minimizing joint destruction and preserving function [9,13,14]. To our knowledge, bilateral postoperative septic arthritis following bilateral quadriceps tendon repair is exceptionally rare, highlighting the unusual nature of this presentation. 

Pyogenic septic arthritis is characterized by infiltration of neutrophils and macrophages, with release of proinflammatory cytokines such as TNF-α and IFN-γ that contribute to cartilage destruction and bone resorption [14]. Patients typically present with acute joint swelling, erythema, warmth, pain with movement, and, in some cases, systemic symptoms such as fever [9]. Laboratory findings, including leukocytosis and elevated ESR and CRP, together with synovial fluid analysis, are essential components of the diagnostic evaluation [13]. In our patient, bilateral knee aspiration, synovial fluid cultures, and intraoperative tissue cultures confirmed MSSA, establishing the diagnosis of bilateral postoperative septic arthritis. However, synovial fluid analysis has limited sensitivity and may yield false-negative results, particularly in early or partially treated infections. Although conventional cultures remain the diagnostic gold standard, newer modalities such as multiplex PCR have demonstrated comparable accuracy while providing more rapid results, although their clinical utility depends on the infecting organism and institutional availability [9].

Management of septic arthritis includes prompt surgical irrigation and debridement, followed by a prolonged course of intravenous antibiotics tailored to culture sensitivities [14]. Delayed or inadequate treatment can result in joint ankylosis, osteomyelitis, or chronic infection [14]. Long-term outcomes largely depend on the timing of intervention and the extent of joint involvement. Although functional recovery is possible with early and aggressive treatment, permanent joint damage remains a significant risk, particularly in older patients and those with comorbidities [14]. Likewise, delayed diagnosis of QTR has been associated with poorer functional outcomes and may necessitate more complex surgical reconstruction, further emphasizing the importance of early recognition and timely intervention [15]. Our patient's need for multiple irrigation and debridement procedures followed by bilateral partial synovectomy underscores the aggressive nature of postoperative septic arthritis and the importance of close postoperative surveillance. 

Despite the absence of many established systemic risk factors for bilateral QTR, our patient had a history of chronic tobacco use and long-term high-dose atorvastatin therapy. Statins are well recognized for causing musculoskeletal adverse effects, ranging from mild myalgias to severe immune-mediated necrotizing myopathy [16,17]. Several case reports have described patients receiving atorvastatin who developed progressive proximal muscle weakness with markedly elevated creatine kinase levels, consistent with statin-induced immune-mediated necrotizing myopathy [18,19]. Additionally, Nesselroade and Nickels reported a case of bilateral QTR in a patient receiving long-term statin therapy, suggesting a possible association between statin use and tendon injury; however, the available evidence is limited to isolated case reports and does not establish causality [20]. Given the limited available evidence, the contribution of chronic tobacco use or long-term atorvastatin therapy to the development of bilateral QTR in our patient cannot be determined. The development of bilateral postoperative septic arthritis following bilateral QTR repair remains exceptionally uncommon and emphasizes the importance of maintaining a high index of suspicion for postoperative infection, even in patients without significant known risk factors. 

## Conclusions

This case illustrates the rare occurrence of bilateral QTR complicated by postoperative septic arthritis in both knees. It emphasizes the importance of early recognition, prompt surgical intervention, and coordinated postoperative care to reduce long-term morbidity and preserve functional outcomes. Notably, this presentation occurred in the absence of typical predisposing factors such as systemic inflammatory disease, immunosuppression, or chronic renal dysfunction. The patient’s history of chronic high-dose atorvastatin use raises the possibility of an underlying medication-related contribution to tendon vulnerability, although further studies are needed to establish this connection. This case highlights the need for clinicians to maintain a high index of suspicion for atypical risk factors in patients presenting with bilateral tendon rupture and to remain vigilant for postoperative infectious complications that may significantly affect recovery.

## References

[1] Pope JD, Bitar YE, Mabrouk A, Plexousakis MP (2026). Quadriceps Tendon Rupture. StatPearls Publishing, Treasure Island, FL.

[2] Matthies NF, Paul RA, Dwyer T, Chahal J, Whelan D (2022). A survey of treatment trends for acute quadriceps tendon ruptures among North American surgeons. Orthop J Sports Med.

[3] Ciriello V, Gudipati S, Tosounidis T, Soucacos PN, Giannoudis PV (2012). Clinical outcomes after repair of quadriceps tendon rupture: a systematic review. Injury.

[4] Camarda L, D'Arienzo A, Morello S, Guarneri M, Balistreri F, D'Arienzo M (2017). Bilateral ruptures of the extensor mechanism of the knee: a systematic review. J Orthop.

[5] Ellanti P, Davarinos N, Morris S, Rice J (2012). Bilateral synchronous rupture of the quadriceps tendon. Ir J Med Sci.

[6] Riley G (2004). The pathogenesis of tendinopathy. A molecular perspective. Rheumatology (Oxford).

[7] Del Buono A, Oliva F, Osti L, Maffulli N (2013). Metalloproteases and tendinopathy. Muscles Ligaments Tendons J.

[8] Momodu II, Savaliya V (2026). Septic Arthritis. https://www.ncbi.nlm.nih.gov/books/NBK538176/.

[9] He M, Arthur Vithran DT, Pan L, Zeng H, Yang G, Lu B, Zhang F (2023). An update on recent progress of the epidemiology, etiology, diagnosis, and treatment of acute septic arthritis: a review. Front Cell Infect Microbiol.

[10] Kim BS, Kim YW, Song EK, Seon JK, Kang KD, Kim HN (2012). Simultaneous bilateral quadriceps tendon rupture in a patient with chronic renal failure. Knee Surg Relat Res.

[11] Pocock CA, Trikha SP, Bell JS (2008). Delayed reconstruction of a quadriceps tendon. Clin Orthop Relat Res.

[12] Langenhan R, Baumann M, Ricart P, Hak D, Probst A, Badke A, Trobisch P (2012). Postoperative functional rehabilitation after repair of quadriceps tendon ruptures: a comparison of two different protocols. Knee Surg Sports Traumatol Arthrosc.

[13] Sajovic M, Nič Ar GL, Dernovš Ek MZ (2009). Septic arthritis of the knee following anterior cruciate ligament reconstruction. Orthop Rev (Pavia).

[14] Kaye AD, Greene D, Alvarez-Amado AV (2024). Pathophysiology and evolving treatment options of septic arthritis: a narrative review. Cureus.

[15] Levinson J, Mixon A (2024). Delayed diagnosis of quadriceps tendon rupture. Cureus.

[16] Mammen AL (2016). Statin-associated autoimmune myopathy. N Engl J Med.

[17] Nazir S, Lohani S, Tachamo N, Poudel D, Donato A (2017). Statin-associated autoimmune myopathy: a systematic review of 100 cases. J Clin Rheumatol.

[18] Bellofatto IA, Sessarego M, Tirandi A (2023). Statin-induced necrotizing autoimmune myopathy with anti-HMGCR antibodies: report of two cases and review of the literature. Case Rep Med.

[19] Paracana B, Amado C, Krowicki J, Monteiro S, Sousa M (2024). Statin-induced necrotizing autoimmune myopathy: a case report and literature review. Cureus.

[20] Nesselroade RD, Nickels LC (2010). Ultrasound diagnosis of bilateral quadriceps tendon rupture after statin use. West J Emerg Med.

